# Dual-Energy Computed Tomography in Stroke Imaging: Value of a New Image Acquisition Technique for Ischemia Detection after Mechanical Thrombectomy

**DOI:** 10.1007/s00062-023-01270-6

**Published:** 2023-03-02

**Authors:** Risto Grkovski, Leyla Acu, Uzeyir Ahmadli, Dominik Nakhostin, Patrick Thurner, Lorenz Wacht, Zsolt Kulcsár, Hatem Alkadhi, Sebastian Winklhofer

**Affiliations:** 1 Department of Neuroradiology, University Hospital Zurich, University of Zurich, Frauenklinikstrasse 10, 8091 Zurich, Switzerland; 2 Institute of Diagnostic and Interventional Radiology, University Hospital Zurich, University of Zurich, Rämistrasse 100, 8091 Zurich, Switzerland; 3 Faculty of Medicine, University of Ljubljana, Vrazov trg 2, 1000 Ljubljana, Slovenia; 4 Department of Radiology, University Medical Centre Maribor, Ljubljanska ulica 5, 2000 Maribor, Slovenia

**Keywords:** Dual energy CT scanner, Cerebrovascular accident, Cerebrovascular occlusion, Virtual noncontrast, Improved stroke detection, Endovascular thrombectomy, Endovascular revascularization

## Abstract

**Objective:**

To assess if a new dual-energy computed tomography (DECT) technique enables an improved visualization of ischemic brain tissue after mechanical thrombectomy in acute stroke patients.

**Material and Methods:**

The DECT head scans with a new sequential technique (TwinSpiral DECT) were performed in 41 patients with ischemic stroke after endovascular thrombectomy and were retrospectively included. Standard mixed and virtual non-contrast (VNC) images were reconstructed. Infarct visibility and image noise were assessed qualitatively by two readers using a 4-point Likert scale. Quantitative Hounsfield units (HU) were used to assess density differences of ischemic brain tissue versus healthy tissue on the non-affected contralateral hemisphere.

**Results:**

Infarct visibility was significantly better in VNC compared to mixed images for both readers R1 (VNC: median 1 (range 1–3), mixed: median 2 (range 1–4), *p* < 0.05) and R2 (VNC: median 2 (range 1–3), mixed: 2 (range 1–4), *p* < 0.05). Qualitative image noise was significantly higher in VNC compared to mixed images for both readers R1 (VNC: median 3, mixed: 2) and R2 (VNC: median 2, mixed: 1, *p* < 0.05, each). Mean HU were significantly different between the infarcted tissue and the reference healthy brain tissue on the contralateral hemisphere in VNC (infarct 24 ± 3) and mixed images (infarct 33 ± 5, *p* < 0.05, each). The mean HU difference between ischemia and reference in VNC images (mean 8 ± 3) was significantly higher (*p* < 0.05) compared to the mean HU difference in mixed images (mean 5 ± 4).

**Conclusion:**

TwinSpiral DECT allows an improved qualitative and quantitative visualization of ischemic brain tissue in ischemic stroke patients after endovascular treatment.

## Introduction

Stroke is the second leading cause of mortality worldwide and an important contributor to disability [1]. Numerous studies have shown the benefit of endovascular mechanical thrombectomy (EVT) in patients with large vessel stroke [2, 3]. Follow-up imaging after the intervention can be performed to assess the extension of the ischemic brain tissue, to rule out intracranial hemorrhage, to evaluate the vessel status and to exclude further potential complications. As this might impact the ongoing treatment as well as the prognosis for the patient, an affordable and easily accessible one stop imaging technique is one of the focuses in stroke imaging and assessment. Dual-energy computed tomography (DECT) can be a helpful diagnostic imaging modality in order to optimize these imaging tasks. Currently there are a number of available DECT techniques of the main vendors, such as: (1) dual-source X‑ray tubes performing simultaneous acquisitions using different tube voltages (e.g., 80–100 kV and 140–150 kV), single-source rapid voltage switching between two tube voltages, a single-source split beam into two different energy spectra and a dual-layer detector with simultaneous data acquisition of the low-energy and high-energy dataset [4–7]. Various DECT applications have been implemented in stroke assessment, for example, in differentiating acute intracranial hemorrhage from contrast staining or calcification [8, 9], visualization of ischemic changes and brain edema [5, 10, 11], prediction of infarction development or hemorrhagic transformation in contrast-enhancing areas [12], and detecting a hyperdense artery sign [13]. Recently, a new technique has been developed, TwinSpiral DECT, a variant of dual spiral DECT, which uses a tin filter and an improved detector enabling low-dose dual-energy scans for optimal spectral separation with a better soft tissue contrast [14]. It acquires two sequential scans in a very short time, one with high and one with low energy immediately after each other.

A recent publication has shown the feasibility of TwinSpiral DECT to differentiate between intracranial hemorrhage and iodine staining with a high accuracy [15]. The aim of the current study was to assess whether TwinSpiral DECT allows an improved visualization of ischemic brain tissue after mechanical thrombectomy.

## Material and Methods

### Patients

All procedures were performed in accordance with local and federal regulations and the Declaration of Helsinki. The study was approved by the local ethics committee (approval number 2018-01212). All patients with an acute ischemic stroke due to middle cerebral artery occlusion (M1 or proximal M2 segment) and consequential mechanical thrombectomy between September 2019 and December 2020 were retrospectively reviewed. Inclusion criteria were a patient age > 18 years and the availability of a TwinSpiral DECT scan within 24 h after thrombectomy and exclusion criteria were an extensive intracranial hemorrhage or materials producing substantial artifacts. A total of 41 patients (18 women, 44%, 23 men, 56%, mean age 73 ± 11.2 years, range 50–93 years) were included in the study analysis.

### Imaging and Postprocessing

**Fig. 1:**
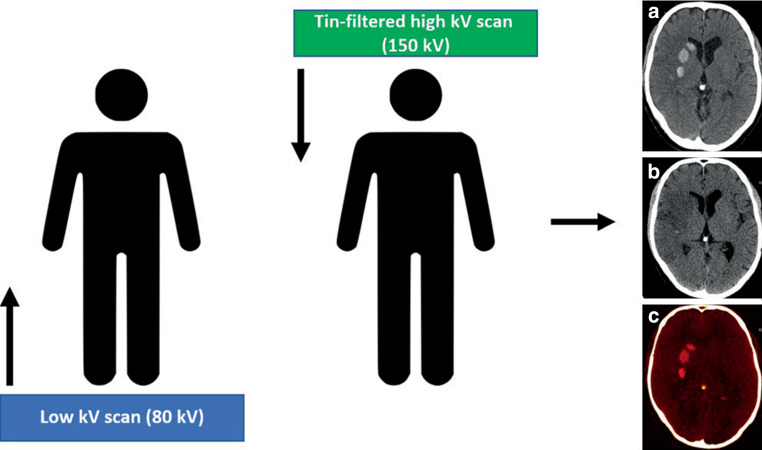
Schematic representation of a single-source TwinSpiral DECT scan and reconstructions. Two consecutive scans at a different energy (low and a high kV scan) are performed consecutively. From these two datasets, conventional mixed CT images (**a**), virtual non-contrast (VNC) images (**b**), and color-coded iodine overlay images (**c**) can be postprocessed

An unenhanced DECT scan was performed in all patients using a single source Twin-Spiral DECT scanner (X.cite, Siemens Healthineers, Forchheim, Germany). Postinterventional follow-up imaging after mechanical thrombectomy is part of a standard routine process for patients after acute ischemic stroke at the Department of Neuroradiology, University Hospital Zurich. For image acquisition, the following parameters were used: Tube voltages were set to 80 kV and tin (Sn) filtered 150 kV (effective reference of 379mA and 318mA, respectively) (Fig. 1). A slice thickness of 1.5 mm, a pitch factor of 0.55 and an incremental acquisition mode with a 64 × 0.6 mm collimation were used. The mean computed tomography dose index volume (CTDIvol) was 43.6 ± 3.7 mGy. Image reconstruction included both virtual non-contrast (VNC) and standard mixed images in axial orientation and an image matrix of 512 × 512 mm, with a slice thickness of 4 mm and an increment of 4 mm. A convolution Kernel Qr40 was used for VNC and a Hr40 for standard mixed images. Standard mixed images, which are used for routine clinical interpretation are weighted images generated from the two energies (80 and 150 kV) resembling a conventional polychromatic single-energy head CT scan with 120 kVp [16–18]. These reconstructions were performed at a dedicated postprocessing workstation (syngo MultiModality Workplace, CT Dual-Energy, Virtual-Unenhanced application, syngo.via, version VB.40 client 4.0, Siemens Healthineers AG, Erlangen, Germany).

### Image Analysis

Image analysis included a quantitative and qualitative image readout of the standard mixed and VNC images. The readout was performed on high-resolution monitors (Flexscan MX 210, Eizo, Ishikawa, Japan), using the picture archiving and communication system (IMPAX EE R20, XVIII SU1, Agfa Healthcare, Bonn, Germany) of the hospital.

#### Qualitative Image Analysis

Qualitative image analysis was performed independently by two experienced board certified and fellowship-trained and independent neuroradiologists (R1 with 9 years of experience in radiology and 6 in neuroradiology, and R2 with 16 years of experience in radiology and 4 in neuroradiology). Both readers were blinded to the image type of reconstruction (VNC or mixed images), clinical patient information and to the results from the second reader. Image windowing was performed individually by each reader. Images were presented to the readers by a third person, who presented patients and their image reconstructions in a random and arbitrary order to both readers.

First, the image quality was evaluated as diagnostic or non-diagnostic. Non-diagnostic criteria included images with severe impairment due to motion or metal artifacts. Second, ischemic changes of the brain parenchyma were assessed by determining the visibility of the contrast between affected hypodense ischemic tissue and unaffected, healthy tissue (1 = good, 2 = moderate, 3 = little contrast, 4 = no contrast). Third, image noise was evaluated in the same regions as previous contrast assessment by grading it into 4 categories (1 = no noise, 2 = little noise with no disturbance of the infarction evaluation, 3 = moderate noise with little disturbance of the infarction evaluation, 4 = severe noise with marked disturbance of the infarction evaluation).

#### Quantitative Image Analysis

Quantitative image analysis was performed by a board certified, fellowship trained neuroradiologist (UA, with 4 years of experience in neuroradiology). The reader opened both standard mixed and VNC images. A region of interest (ROI) was placed in the affected area and was mirrored in the unaffected area on the contralateral healthy hemisphere for each image reconstruction separately. The ROI size was at least 1 cm^2^. Average Hounsfield units (HU) and the standard deviation (SD) were noted for each ROI in both VNC and standard mixed images.

## Statistical Analysis

Descriptive statistics are provided as mean ± standard deviation (range) for continuous and frequencies (*n*) and as median and range values for categorical variables. The Shapiro-Wilk test was performed to investigate for normality distribution.

Cohen’s κ‑coefficients were calculated to evaluate the inter-rater agreement regarding the assessment of contrast and image noise in VNC and mixed images. The κ values below 0.4 were interpreted as poor and between 0.41–0.75 as fair to good according to criteria originally proposed by Landis and Koch [19].

Comparison of qualitative scores (contrast and image noise) between VNC and mixed images was conducted using the pairwise Wilcoxon signed rank test to assess for significant differences.

For quantitative analysis, a statistical significance of mean in differences of HU values for each reconstruction modality was determine using the paired Student’s t‑test. A *p*-value < 0.05 was considered statistically significant. Statistical analyses were conducted using commercially available software (IBM SPSS Statistics, Version 26.0, IBM Corp. Armonk, NY, USA and Microsoft Excel, Microsoft Corporation. (2019). Microsoft Excel. Retrieved from https://office.microsoft.com/excel).

## Results

### Inter-Rater Agreement

The inter-rater agreement regarding the contrast and qualitative image noise between R1 and R2 was low (k < 0.4; *p* < 0.0001); hence qualitative statistical results are presented for each reader separately.

### Qualitative Results

**Fig. 2:**
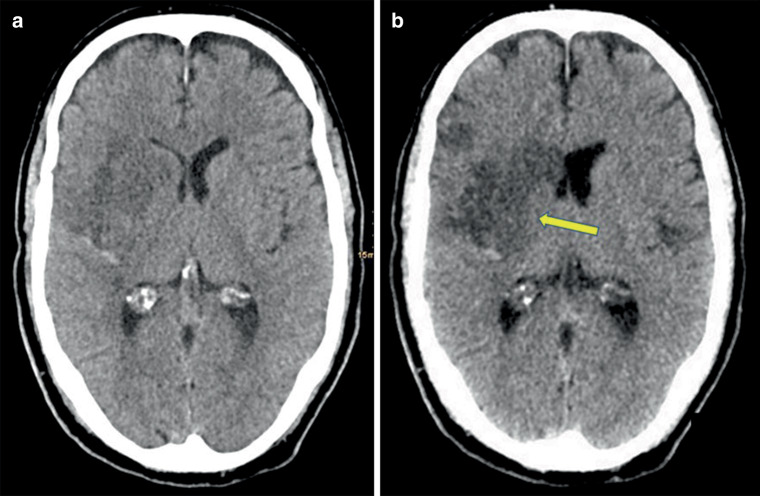
A patient with a right Sylvian artery ischemic stroke. **a** Standard mixed images and **b** virtual non-contrast images (VNC) reconstructed from a TwinSpiral DECT acquisition. Ischemic hypodense area of infarction is better visible on the VNC (*yellow arrow*) compared to the standard mixed images. It shows better contrast and larger impacted area

**Fig. 3:**
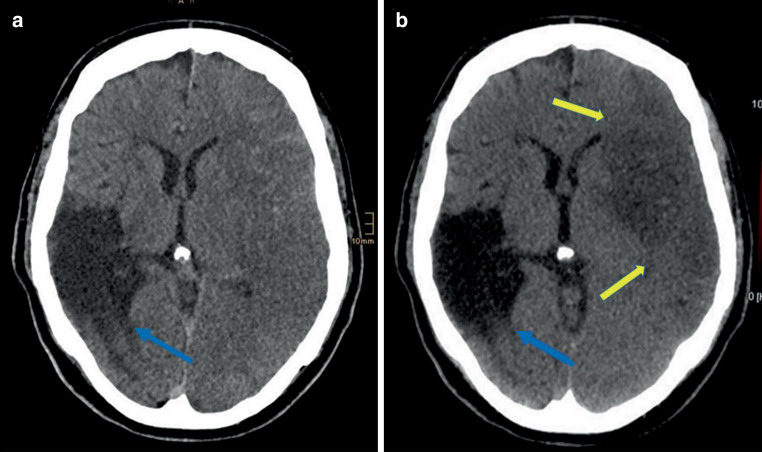
**a** Standard mixed images and **b** virtual non-contrast images (VNC). Note the old right hemispheric infarction is clearly visible in both standard mixed and VNC images (*blue arrow*). However, the acute hypodense ischemic area on the contralateral left hemisphere is much better differentiated on the VNC images (*yellow arrows*) compared to the standard mixed images

**Fig. 4:**
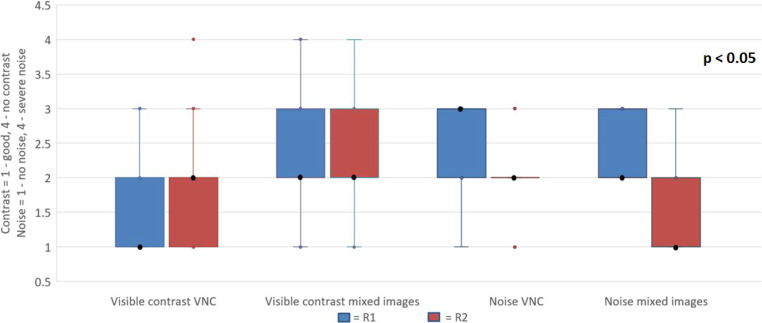
Qualitative evaluation of image contrast and noise. Virtual non-contrast images (VNC) demonstrated better contrast, while also exhibiting increased noise

Both readers rated the overall image quality as diagnostic in all cases and reconstructions. The contrast score was significantly better in VNC images compared to mixed images for both readers R1 (VNC: median 1 (range 1–3), mixed: 2 (range 1–4), *p* < 0.05) and R2 (VNC: median 2 (range 1–3), mixed: 2 (range 1–4), *p* < 0.05) (Figs. 2, 3 and 4).

The qualitative image noise was significantly higher in VNC images compared to mixed images for both readers R1 (VNC: median 3 (range 2–3); mixed: 2 (range 2–3), *p* < 0.05) and R2 (VNC: median 2 (range 1–3); mixed: 1 (range 1–3), *p* < 0.05) (Fig. 4).

### Quantitative Image Analysis

**Fig. 5:**
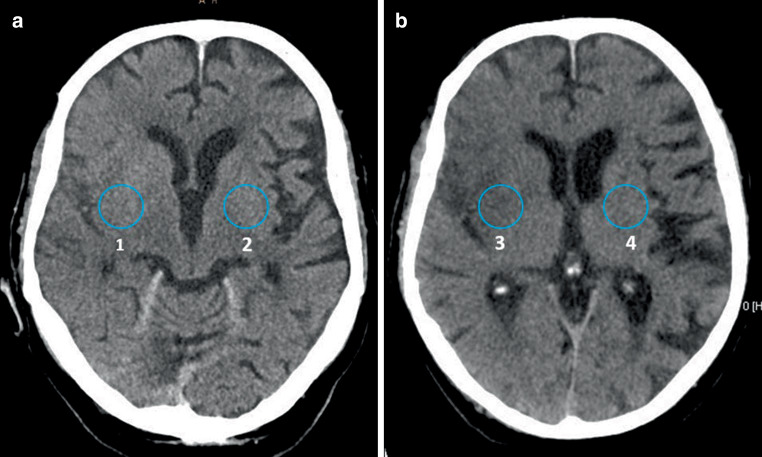
A patient with a right Sylvian artery acute ischemic stroke. Hounsfield units (HU) measurements on the right (affected) and contralateral left (unaffected) side. **a** Standard mixed images: region of interest (ROI) 1—39 HU, ROI 2—42 HU, **b** virtual non-contrast images (VNC): ROI 3—25 HU, ROI 4—33 HU. After the subtraction of values for each image modality, the difference in the VNC images (factor of 8) is bigger compared to the standard mixed images (factor of 3)

**Fig. 6:**
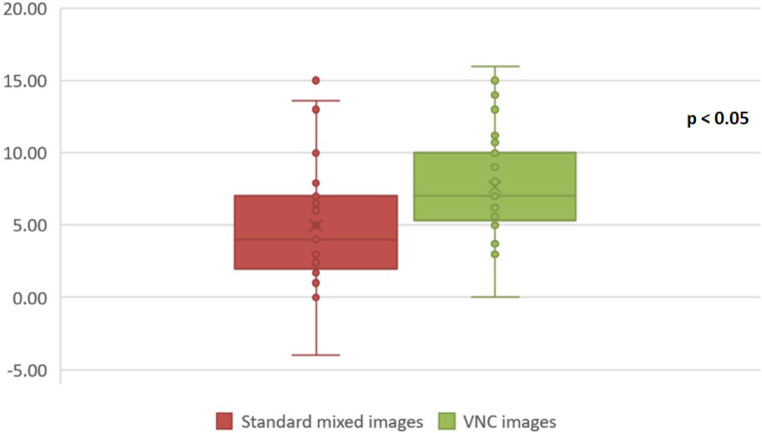
Mean Hounsfield units (HU) difference from region of interest (ROI) measurements between ischemia and reference tissue in the standard mixed images and virtual non-contrast images (VNC)

Mean HU were significantly different between the infarcted tissue and the reference healthy brain tissue on the contralateral hemisphere in VNC (infarct 24 ± 3 HU, reference 31 ± 2 HU) and mixed images (infarct 33 ± 5 HU, reference 38 ± 3 HU) (*p* < 0.05, each). The mean HU difference between VNC ischemia and VNC reference (mean 8 ± 3 HU) was significantly higher (*p* < 0.05) compared to the mean HU difference in mixed images in ischemia and reference tissue (mean 5 ± 4) (Figs. 5 and 6).

## Discussion

This study investigated the value and feasibility of a new DECT technique: TwinSpiral DECT, for an improved ischemic stroke detection and visualization. Our results suggest that TwinSpiral DECT is a feasible method to be performed in patients after mechanical thrombectomy with an improved visualization of the ischemic tissue in VNC compared to the standard mixed images; however, despite our paper focusing on patients post-EVT, as DECT is primarily performed in patients after EVT, the better contrast visibility between healthy and ischemic tissue could be implied in initial stroke assessment as well. Thus, the next step would be a study in which this DECT technique would be applied for the initial imaging in the acute ischemic stroke setting (i.e. before EVT) to assess the benefit of VNC images for the detection of ischemic brain tissue and therefore for a potentially more accurate clinical prognosis and optimal therapeutic management.

By comparing the qualitative values of each individual reader, the VNC images from the tested DECT demonstrated the affected ischemic brain tissue to be significantly more hypodense than in standard mixed images, although with a slight increase of noise. The infarcted areas were better visible in VNC images compared to standard mixed images. Measurements of HU and comparing the values of VNC and standard mixed images showed a markedly increased difference in radiodensity between ischemic and healthy brain tissue, thus allowing an improved delineation and assessment of ischemic stroke.

Detecting ischemic changes post-EVT is just as important as pre-EVT. The distant embolization of the affected vessel and infarcts in new vascular territories are both associated with worse prognosis due to newly developed ischemic regions [20, 21] and impairment of collateral blood flow to already affected areas [22]. Prompt treatment, either by EVT or intra-arterial thrombolysis, is important for favorable clinical outcome and prevention of further complications [23].

The results of our study are comparable to results from Gariani et al. [10]. They have shown to be able to separate ischemic from non-ischemic tissue with major advantages in DECT by using VNC compared to non-contrast CT weighted sum images (NCCT). Another study showed superiority of VNC images compared to conventional images (using a dual-layer spectral CT) in ischemic infarct delineation [24], as well as in detection of ischemic changes in posterior fossa [25]; however, both studies were performed on a DECT acquisition technology other than TwinSpiral DECT. In addition, previous studies have also reported a higher noise in both VNC [26] and virtual monoenergetic imaging [27, 28], which was also observed in our study. Nevertheless, the increased image noise seems not to have a major impact on the detection of hypodense ischemic brain tissue.

The first concept of DECT was mentioned by Hounsfield in 1973, when he described acquiring two separate images (at 100 kV and the other at 140 kV) at the same slice, in order to differentiate iodine from calcium based on different atomic numbers [29]. This required two scans for a single series of images, thus approximately doubling the radiation dose. Recently, a new DECT image technique was developed, TwinSpiral DECT, enabling two scans at low-kV and high-kV data sets integrated into one single acquisition, without an increase in radiation dose. This method does not require dual source, dual layer, or rapid energy switching ability. Instead, a tin filter provides powerful spectral separation [30, 31], while an improved detector used herein enables high-quality imaging at a high temporal resolution in low dose or low signal imaging [14], with utilization of advanced (semi-)automated postprocessing algorithms enabling facilitated routine-ready solutions. As such, it may be applied by a broader pool of health institutions, with less costs than other high-end DECT scanners.

One of the drawbacks of the TwinSpiral DECT technique could be its susceptibility to motion artefacts, such as in agitated patients, where the movement of the patient between the low kV and the high kV scan could potentially compromise the quality of DECT images. In our series, all of the included studies demonstrated diagnostic image quality. No major motion artifacts were seen, which also reflects our personal clinical experience from daily routine with this new dual energy technique; however, even in the case of patient movement in one of the two scans, the second scan could still be used for diagnostic purposes (even though the dual-energy information might get lost). Furthermore, it is important to note that the mean radiation dose was within the range of a normal single-energy head CT scan and in the range of the diagnostic reference levels (DRLs) and achievable doses (ADs) in the USA [32].

In addition to the above investigated application, the TwinSpiral DECT technique might also provide further already established benefits of a DECT, not only in detecting ischemic changes, but differentiating between hemorrhage [15], iodine, calcifications and metal artifacts, without the need for multiple scans [33, 34]. Furthermore, this study might open new frontiers in stroke research, as to evaluate the technique’s ability for ischemic stroke in the posterior circulation or for an improved stroke detection in initial patient presentation.

Although there was somewhat of a discrepancy between inter-reader agreement in qualitative image analysis in our study, each reader represented a separate control, and what is most important, both readers were able to detect significant changes in contrast between VNC and standard mixed images, a key finding in ischemic brain tissue.

Limitations of our study were the relatively small retrospective study size and blinding, which was not completely possible, as experienced readers could easily recognize the type of DECT imaging reconstruction. Furthermore, qualitative image analysis could be done by ASPECTS, which, in future studies including more patients, might be used to assess prognosis in pre-EVT in post-EVT patients.

## Conclusion

Our study demonstrated that TwinSpiral as a new DECT technique allows an improved qualitative and quantitative visualization of ischemic brain tissue. This indicates that TwinSpiral DECT might be a valuable tool for imaging of ischemic stroke patients after endovascular treatment.
